# Diffuse-Type Tenosynovial Giant Cell Tumour Involving Bone Masquerading as Langerhans Cell Histiocytosis

**DOI:** 10.1155/2022/1992541

**Published:** 2022-09-15

**Authors:** Florence M. F. Cheung, Timothy Y. C. So, Tony H. T. Sung, Ying-Lee Lam

**Affiliations:** ^1^Department of Clinical Pathology, Gleneagles Hospital Hong Kong, Wong Chuk Hang, Hong Kong; ^2^Musculoskeletal Tumour Centre, Gleneagles Hospital Hong Kong, Wong Chuk Hang, Hong Kong; ^3^Radiology Department, Gleneagles Hospital Hong Kong, Wong Chuk Hang, Hong Kong; ^4^Department of Orthopaedics and Traumatology, Queen Mary Hospital, Pok Fu Lam, Hong Kong

## Abstract

We presented a case of diffuse-type tenosynovial giant cell tumour (DTSGCT) of foot masquerading as Langerhans cell histiocytosis. Preliminary diagnosis by needle biopsy was difficult due to the major involvement of bones and the overshadowing effect of the accompanying Langerhans cells. The complete curettage specimen with relevant immunohistochemistry and molecular tests made the final diagnosis of DTSGCT possible. The biomolecular mechanism for the masquerading phenomenon was explained by CSF1 overexpression in the neoplastic cells attracting migration and proliferation of CSF1R-positive Langerhans cells.

## 1. Introduction

An accurate and prompt diagnosis of symptomatic bone tumours requires a multidisciplinary team approach [1]. This includes a well-planned biopsy at dedicated medical centre with prior input from the orthopedic surgeon, radiologist, pathologist, and oncologist. Needle biopsy if properly conducted is preferred over open biopsy for minimal wound contamination. However, sampling limitations and mimickers of various bone lesions do pose difficulties in making an accurate diagnosis. We present a case we encountered recently that illustrates such difficulties.

## 2. Case Presentation

**Figure 1:**
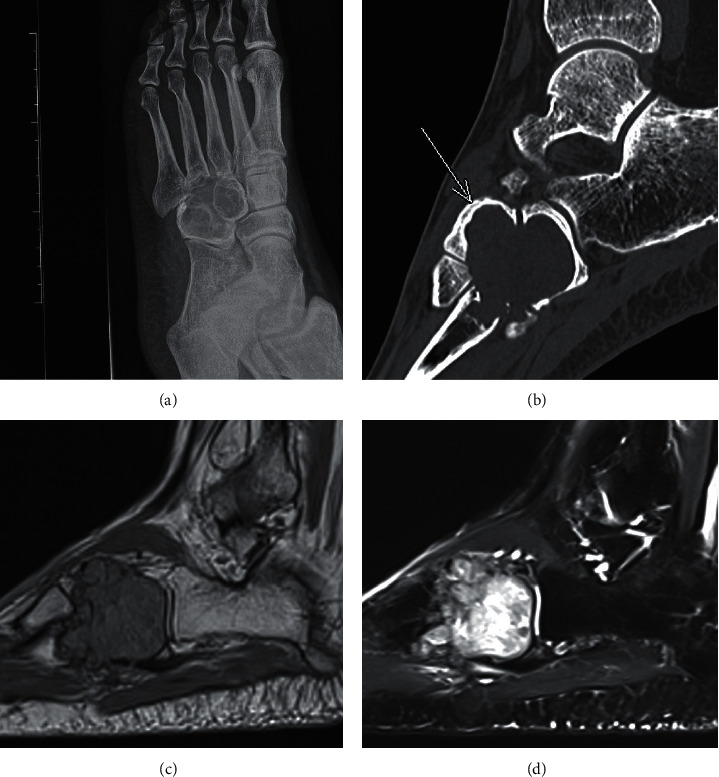
(a) Plain X-ray of left foot demonstrated an expansile lytic lesion in cuboid and lateral cuneiform. (b) CT scan highlighted its well-defined margin and cortical scalloping of lateral cuneiform (arrowed). (c) MRI showed PD-intermediate signal of the lobulated lesion with distal cortical perforation and (d) hyperintense signal on T2.

A 32-year-old man presented half-a-year ago with pain over his left foot for several months. He noticed swelling over the same site since teenage years with no medical consultation. Physical examination revealed a firm swelling over his left foot lateral aspect. Plain X-ray and CT scan (Figures 1(a) and 1(b)) showed a multilobulated expansile osteolytic lesion in the left cuboid and lateral cuneiform bones. It crossed the tarsal and tarsal-metatarsal joints with early extension to the base of the 3^rd^, 4^th^, and 5^th^ metatarsal, totally measuring 3.0 × 3.5 × 3.5 cm. Cortical scalloping and erosions were seen in these bones as well as focal sclerotic border. MRI demonstrated PD-intermediate and T2-hyperintense signal in the lesion matrix (Figures 1(c) and 1(d)). A cortical defect was present at plantar aspect of the cuboid with tumour extrusion into the plantar soft tissue. ^18^F-FDG PET showed marked hypermetabolism over peripheral aspect of the lesion (SUVmax 16.1). No hypermetabolic nodal or distant metastasis was detected. The radiological features suggested a locally aggressive primary bone tumour.

**Figure 2:**
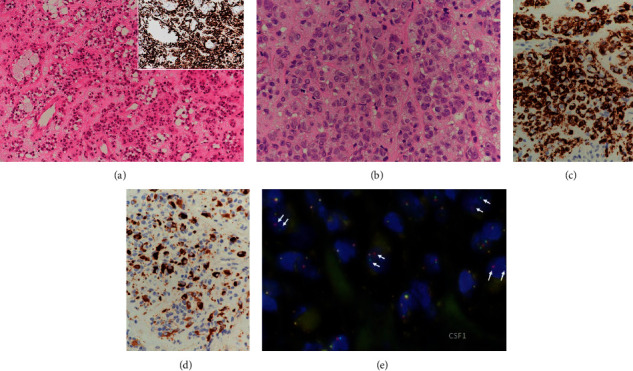
(a) Needle biopsy of cuboid lesion showed clusters and cords of pinkish epithelioid cells in a fibromyxoid stroma (H&E x40). The insert showed numerous CD1a-positive cells on immunohistochemistry (IHC x40). (b) The curettage specimen showed cellular clusters of pinkish epithelioid cells with open nuclei (H&E x400). (c) Majority of epithelioid cells are Langerin-positive Langerhans cells (IHC x400). (d) Scattered cells are Clusterin-positive tumour cells (IHC x400). (e) FISH showed scattered nuclei with break-apart green and red signals of CSF-1 (arrowed).

Needle biopsy was done and showed tumour tissue characterized by clusters and cords of pinkish epithelioid cells in a fibromyxoid stroma (Figure 2(a)). The epithelioid cells have central to eccentric open nuclei with distinct nucleoli. There was no significant nuclear pleomorphism or frank malignancy. A prominent chronic inflammatory cell infiltrate including lymphocytes, foamy macrophages, and histiocytes was noted in the background. No multinucleated giant cells were seen. Initial immunohistochemistry (IHC) showed most cells in the lesion were positive for endothelial markers and histiocytic markers. Differential diagnosis of epithelioid haemangioendothelioma was excluded by the negative IHC for CAMTA1 and TFE3. Further IHC demonstrated numerous CD1a (Figure 2(a), insert) and Langerin-positive Langerhans cells, while H3.3G34 W and H3K36 M for giant cell tumour of bone and chondroblastoma, respectively, were negative. Thinking along the line of a primary bone tumour, diagnosis of Langerhans cell histiocytosis was suggested, but with reservation due to the paucity of eosinophils and the prominent fibromyxoid stroma. Complete curettage of the lesion and cementation was then performed. The curettage specimen showed fragments of fibronodular tumour tissue with patchy necrosis and well-defined borders. There were cords and clusters of pinkish epithelioid cells similar to those seen in the needle biopsy (Figure 2(b)). Most of these cells were CD1a- and Langerin-positive on IHC (Figure 2(c)). In addition, there were prominent pseudoalveolar spaces, focal chondroid metaplasia, aggregates of multinucleated giant cells, and scattered Desmin-positive dendritic cells. These features were compatible with a diffuse-type tenosynovial giant cell tumour (DTSGCT), being supported by IHC for Clusterin [2] which stained a minor population of the epithelioid cells (Figure 2(d)) and cells lining the pseudoalveoli. This diagnosis was further confirmed by fluorescent in situ hybridization (FISH) demonstrating colony stimulating factor 1 (CSF1) translocation in scattered cells using a break-apart probe (Figure 2(e)). The patient remained unremarkable 3 months after surgery and would be monitored for local recurrence.

## 3. Discussion

DTSGCT of soft tissue, whether intraarticular or extraarticular, is often symptomatic and prone to local recurrence if incompletely excised. DTSGCT involving bones of the foot rarely occurs, and some cases have been reported in the literature [3, 4]. The present tumour most likely originated from the tarsal synovial joint with secondary bulky involvement of the adjacent tarsal and metatarsal bones. Besides the major intraosseous location, needle biopsy interpretation was compounded by the numerous reactive Langerhans cells, raising the suspicion of Langerhans cell histiocytosis. However, the paucity of eosinophils and the prominent fibromyxoid stroma were features unusual for this diagnosis. The long clinical history offered by the patient, plus focal sclerotic border on plain-X-ray and lack of frank malignancy by microscopy, pointed to a locally aggressive slow-growing tumour. This warranted complete curettage as the surgical treatment of choice. The pathological features of the curettage specimen were classical of diffuse-type tenosynovial giant cell tumour. This diagnosis was supported by IHC for Clusterin confirming the presence of synovial cells and FISH confirming the translocation of CSF1 in tumour cells. AS DTSGCT is prone to local recurrence, follow-up monitoring of this patient was also advised.

On retrospect, the prominent Langerhans cell proliferation could be explained by CSF1 translocation in tumour cells and their subsequent overexpression of CSF1 protein [5]. The latter attracted the migration and proliferation of Langerhans cells together with other dendritic cells which possessed the cell surface protein CSF1R [6]. This contributed to the “landscaping effect” by macrophages and various dendritic cells so commonly seen in tenosynovial giant cell tumours.

## 4. Conclusion

We would like to raise awareness for the possible major bone involvement by DTSGCT mimicking a primary bone tumour and the exuberant reactive Langerhans cell proliferation mimicking Langerhans cell histiocytosis. Our case is unique with these two features occurring together, thus making diagnosis on the needle biopsy difficult. Although the underlying biomolecular mechanism is now known, the masquerading effect of Langerhans cells in DTSGCT has not been highlighted before in the medical literature and therefore is worth emphasizing.

## Data Availability

The data that support the findings of this study are available from the corresponding author upon reasonable request.
